# Screening of Phenanthroquinolizidine Alkaloid Derivatives for Inducing Cell Death of L1210 Leukemia Cells with Negative and Positive P-glycoprotein Expression

**DOI:** 10.3390/molecules24112127

**Published:** 2019-06-05

**Authors:** Jana Kubíčková, Katarína Elefantová, Lucia Pavlikova, Martin Cagala, Mário Šereš, Peter Šafář, Štefan Marchalín, Kamila Ďurišová, Viera Boháčová, Zdena Sulova, Boris Lakatoš, Albert Breier, Petra Olejníková

**Affiliations:** 1 Institute of Biochemistry and Microbiology, Faculty of Chemical and Food Technology, Slovak University of Technology in Bratislava, Radlinského 9, 812 37 Bratislava, Slovakia; jana.kubickova@stuba.sk (J.K.); katarina.elefantova@stuba.sk (K.E.); kamila.durisova@stuba.sk (K.Ď.); boris.lakatos@stuba.sk (B.L.); 2 Institute of Molecular Physiology and Genetics, Centre of Bioscience, Slovak Academy of Sciences, Dubravska Cesta 9, 840 05 Bratislava, Slovakia; lucia.pavlikova@savba.sk (L.P.); martin.cagala@savba.sk (M.C.); mario.seres@savba.sk (M.Š.); viera.bohacova@savba.sk (V.B.); zdena.sulova@savba.sk (Z.S.); 3 Institute of Organic chemistry, Faculty of Food and Chemical Technology, Slovak University of Technology in Bratislava, Radlinského 9, 812 37 Bratislava, Slovakia; peter.safar@stuba.sk (P.Š.); stefan.marchalin@stuba.sk (Š.M.)

**Keywords:** L1210 cells, P-glycoprotein, multidrug resistance, phenanthroquinolizidine alkaloids, apoptosis

## Abstract

We describe the screening of a set of cryptopleurine derivatives, namely thienoquinolizidine derivatives and (epi-)benzo analogs with bioactive phenanthroquinolizidine alkaloids that induce cytotoxic effects in the mouse lymphocytic leukemia cell line L1210. We used three variants of L1210 cells: i) parental cells (S) negative for P-glycoprotein (P-gp) expression; ii) P-glycoprotein positive cells (R), obtained by selection with vincristine; iii) P-glycoprotein positive cells (T), obtained by stable transfection with a human gene encoding P-glycoprotein. We identified the most effective derivative **11** with a median lethal concentration of ≈13 μM in all three L1210 cell variants. The analysis of the apoptosis/necrosis induced by derivative **11** revealed that cell death was the result of apoptosis with late apoptosis characteristics. Derivative **11** did not induce a strong alteration in the proportion of cells in the G_1_, S or G_2_/M phase of the cell cycle, but a strong increase in the number of S, R and T cells in the subG_1_ phase was detected. These findings indicated that we identified the most effective inducer of cell death, derivative **11**, and this derivative effectively induced cell death in S, R and T cells at similar inhibitory concentrations independent of P-gp expression.

## 1. Introduction

While much attention has been paid to finding effective anticancer agents, these discoveries remain important for future improvements in cancer treatment. In order to find novel derivatives of naturally occurring compounds with potentiated cytotoxic effects, neoplastic cells are important for rational anticancer drug development [1]. Moreover, neoplastic cells can develop pleiotropic cell resistance based on multifactorial inducement [2]. Such resistance leads to reduced drug-induced cytotoxicity, which enables cells to escape drug-induced death. This cell survival points to the necessity to include knowledge about the induction and/or development of this resistance when designing and preparing drugs with the potential for use as novel anticancer therapies [3].

Plant alkaloids represent a large but well-defined group of nitrogen-containing substances that have been used in both traditional and alternative medical practice to treat many diseases because of their wide range of biological activities. Currently, plant alkaloids are at the forefront of therapeutic discovery and serve mainly as suitable structures for the derivatization and development of new drugs with potential use in modern medicine. The derivatization of well-known natural compounds/plant metabolites with a wide range of biological activities is a promising way for focusing on their specific effectiveness [4]. A rich source of plant alkaloids with a wide range of biological activities is found in the *Leguminosae* family. Cryptopleurine, a phenanthroquinolizidine alkaloid, was isolated from *Cryptocarya* and *Boehmeria* species [5] as a compound with potent antiviral [6], anti-inflammatory [7] and antiproliferative activity [8,9]. It is representative of natural compounds with a common pentacyclic structure such that the phenanthrene ring is conjugated with quinolizidine. Phenanthroquinolizidines have gained renewed attention because of their described mode of action, which differs from that of currently used drugs [10]. Many potential biological targets of phenanthroquinolizidines have been reported. The antiproliferative action of phenanthroquinolizidines seems to be associated with the downregulation of cell cycle regulatory proteins such as cyclin and cyclin-dependent kinases [11]. Several other quinolizine structures have been reported as inhibitors of DNA topoisomerase I activity such that the cell cycle is arrested at the G0/G1 phase [12].

In the present paper, we report the cytotoxic effects of a set of cryptopleurine derivatives (thienoquinolizidine derivatives and (epi-)benzo analogs with bioactive phenanthroquinolizidine alkaloids) obtained by organic synthesis [13,14] on the lymphocytic leukemia cell line L1210. When looking for new active structures with potential antileukemic activity, it is important to think about the potential risks of multidrug resistance (MDR) development. The most often observed mechanism of neoplastic cell resistance stems from the enhanced expression or activity of plasma membrane efflux pumps, classified as ABC transporters [15], that are able to eliminate various unrelated chemicals with diverse structures from intracellular space. The overexpression of these efflux pumps is one of the molecular-based causes of MDR. Therefore, it is important to test new, active molecular structures as substrates for efflux pumps. The overexpression of P-glycoprotein (P-gp), the most frequently occurring drug efflux pump of the plasma membrane (an ABCB1 member of the ABC transporter gene family), in neoplastic cells is generally accepted as the molecular mechanism behind the dramatically reduced cell sensitivity to a well-defined group of anticancer drugs known as P-gp substrates [16]. Therefore, we aimed to test for the cytotoxic effects of thienoquinolizidine derivatives and the (epi-)benzo analogs of bioactive phenanthroquinolizidine alkaloids on cells with and without expression- or drug-induced P-gp efflux activity. The biological model used in the current study is based on three variants of L1210 cells: parental drug-sensitive cells that do not express P-gp (S) and two drug-resistant P-gp-positive cell variants obtained by either S cell adaptation to vincristine (R) or transfection of S cells with the human gene encoding P-gp (T) [17].

## 2. Results

### 2.1. Characterization of L1210 Cell Variants

The cytotoxic effects of a newly prepared set of quinolizidine derivatives QDs were evaluated on three variants of L1210 cells that differed in their expression of P-gp. These variants included parental P-gp-negative (S) cells and two P-gp-positive cell variants obtained either by selection with vincristine (R) [18] or by transfection with a gene encoding P-gp (T) [17]. We detected massive amounts of P-gp mRNA and protein by RT-PCR and Western blotting, respectively, in P-gp-positive R and T variants. In contrast, the detection of P-gp mRNA expression and protein levels gave only weak (if any) signals in S cells [17]. Moreover, we also demonstrated that the P-gp efflux activities that led to decreased calcein retention within R and T cells were lacking in S cells. Consistent with this finding, R and T cells were much less sensitive to P-gp substrates, such as VCR, doxorubicin, and mitoxantrone, than S cells [19]. P-gp was detected by immunofluorescence confocal microscopy in R and T cells predominantly in the plasma membrane [20]. In contrast, no immunoreactive materials were visible in S cells. All these features are consistent with previously published data [17,19,21,22] and were periodically controlled in our laboratory in the experiments described in the current paper. Taken together, the characteristics of S, R and T cells make them a suitable cell model for studying the differences between the responses of P-gp-negative and P-gp-positive leukemia cells to various chemicals.

### 2.2. Characterization of QD Derivatives

Thirteen isomeric (epi)-thieno analogs of phenanthroquinolizidine (TQD, denoted as **cis10Aa**, **cis10Ab**, **trans10Aa**, trans**10Ab**, **trans10Bb**, **11Ba**, **11Bb**, **11Aa**, **11Ab**, **15a**, **15b**, **18Aa** and **18Ab**) with structural motifs similar to those of naturally occurring cryptopleurine and hydroxycryptopleurine were used in this study (Table 1).

The analogs were synthesized from L-2-aminoadipic acid and the corresponding thiophen-2-carbaldehyde or thiophen-3-carbaldehyde. L-2-aminoadipic acid was used as a source of chirality as well as for the nitrogen necessary for further applications, specifically for the synthesis of these types of compounds. *N*-methylthienoquinolizinium iodides **A4**, **B9**, and **B10** were obtained by *N*-alkylation of the corresponding thienoquinolizidines **10Bb**, **11Ba**, and **11Bb** [23]. The asymmetric synthesis of (epi)-benzo analogs of the phenanthroquinolizidine bioactive alkaloids (-)-cryptopleurine and (-)-(15R)-hydroxycryptopleurine **5a**, **5b**, **6a**, **7a**, **7b**, **9**, **10** and **11** (BQD) (Table 1) was achieved from enantiopure L-2-aminoadipic acid and benzaldehyde [14]. Details about the synthesis, accepted structure and characterization are published elsewhere [13,14,23].

### 2.3. Effectiveness of QDs to Induce Cell Death of S, R and T Cells

All derivatives were assayed for their cytotoxic effects on leukemia cells. The IC_50_ (median of lethal concentration) values were calculated from growth inhibition curves and are presented in Table 2. Our results show that a higher cytotoxic effect was induced with the BQD derivatives compared to the TQD derivatives. In the TQD group, only derivative **11Ba** was effective on P-gp-negative S cells, and no measurable cytotoxic effect was found in R and T cells at derivative concentrations exceeding 100 μΜ. This finding was used to predict the resistance of P-gp-positive cell variants against **11Ba** (Table 2).

In the BQD group, four effective derivatives were identified; among them, cell death was more effectively induced in P-gp-negative S cells, compared to P-gp positive R and T cells, by derivative **6b** (the IC_50_ values were 192 μΜ for S cells and 385 μΜ for both R and T cells). Derivative **10** seems to be more effective in S than in R and T cells but only at much higher concentrations (the IC_50_ value was 490 μΜ). In contrast to previously tested derivatives, two BQD derivatives, **9** and **11**, induced almost similar levels of cell death on all three cell variants. Moreover, these derivatives were found to be very effective, with IC_50_ values of approximately 72–82 μΜ for derivative **9** and 13 μΜ for derivative **11** (Table 2). Due to their efficacy, both derivatives were assayed on the human acute myeloid leukemia SKM-1 and MOLM-13 cells, and their respective IC_50_ values were similar to those obtained for S, R and T cells, i.e., approximately 60–90 μΜ for derivative **9** and 10–15 μΜ for derivative **11**. In our previous paper [14], the cytotoxic effects induced by derivatives **9** and **11** were tested with two non-neoplastic cell lines (hamster kidney fibroblast cell line BHK-21 and African green monkey kidney fibroblast-like cell line VERO). Derivative **11** reached the IC_50_ value in the higher concentration range of 213 µM for BHK-21 cells and 266 µM for VERO cells. Neither cell line was affected by derivative 9. Thus, it could be concluded that these two derivatives induced more effective cell death in neoplastic cells than in normal cells. The best antiproliferative activity found in S, R and T cells was induced by derivative **11**, and this effect was not influenced by the presence of P-gp in either the R or T cells. Therefore, additional studies of several features associated with the cell death of S, R and T were focused on derivative **11**.

### 2.4. Effectiveness of Derivative 11 on Expression and Efflux Activity of P-gp in R and T Cells

**Figure 1:**
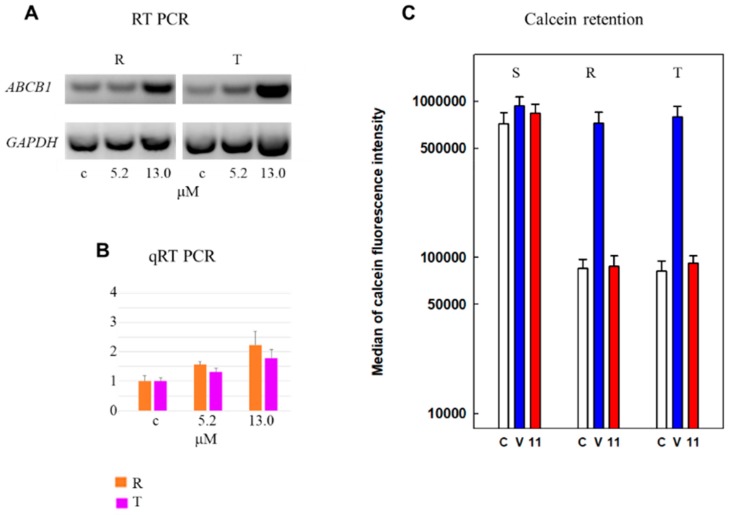
Effect of derivative **11**, applied at concentrations of 0.0 (c), 5.2 and 13.0 μM, on the expression and activity of P-gp. (**A)**: Cell contents of ABCB1 gene transcripts encoding P-gp by RT-PCR (reverse transcription polymerase chain reaction). The results are representative of three independent measurements of triplicate experiments, and transcripts for GAPDH were used as housekeepers. (**B**): Quantification of mRNA content by qRT-PCR. Data represent the mean ± S.E.M. from three independent measurements. The amount of detected PCR product in R cells incubated in the presence of derivative **11** at a concentration of 13.0 μΜ differed significantly from the corresponding value in the absence of substance (c) at the level of p<0.05. Other results did not fulfill the criteria for statistical significance. (**C**): Median values of calcein retention in S, R and T cells in the presence or absence of verapamil (V, 10.0 μM) and derivative **11** (11.0, 13.0 μM). Median values were obtained from corresponding cytometry histograms of calcein fluorescence using BD-Accuri C6 software. Verapamil at this concentration fully restores calcein retention in R and T cells to the extent characteristic of S cells [17]. Data represent the mean ± S.E.M. from three independent measurements. Median values registered for R and T cells in the absence of inhibiting compounds or in the presence of derivative **11** differ significantly (at least on the level of p < 0.02) from median values obtained either from S cells independent of the presence of verapamil) or for R and T cells obtained in the presence of verapamil.

Derivative **11** induced a visible increase in transcripts encoding P-gp in R and T cells, namely, at a concentration of 13 μM, which is equal to that for inducing the IC_50_ value (Figure 1A). This amount was quantified by qRT-PCR, which revealed an approximate doubling of the cellular amount of this transcript (Figure 1B). The transport activity of P-gp was monitored by the retention of the calcein originating from calcein-AM (a P-gp substrate) after esterified carboxylic group liberation with intracellular esterases [20,24]. In S cells, we observed a massive retention of calcein within the cells that could not be influenced by verapamil (a known P-gp inhibitor) or derivative **11** (Figure 1C). In contrast, retention of calcein in both P-gp positive cell variants (R and T) was less pronounced, and could be achieved to an extent similar to that in S cells by verapamil. Contrary to verapamil, derivative **11** did not influence the retention of calcein in R and T cells.

### 2.5. Measurements of the Effectiveness of Derivative 11 to Induce Cell Death Based on FITC-Annexin V and Propidium Iodide Double Staining

**Figure 2:**
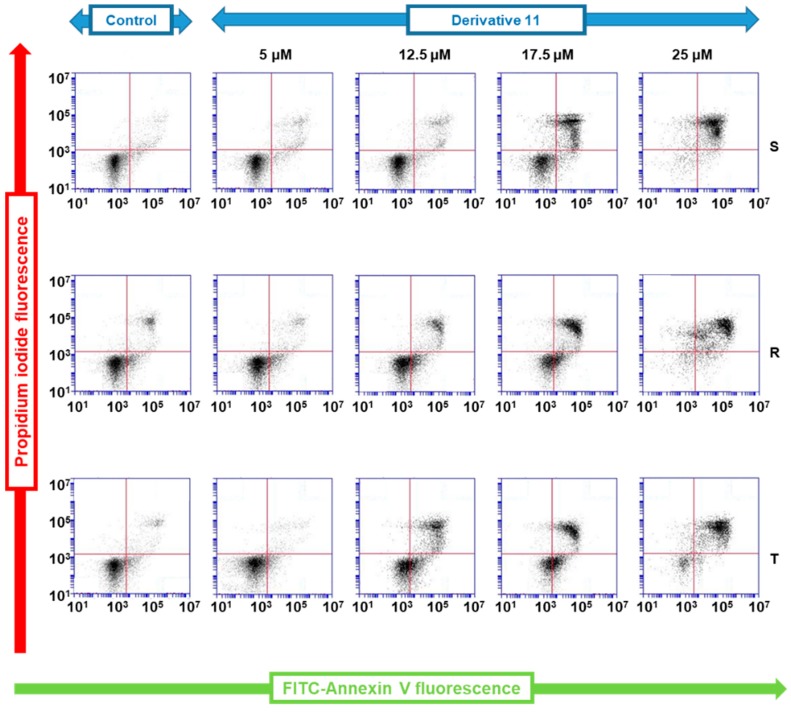
Measurement of S, R and T cell proportions during apoptosis and necrosis as measured using an FAV/PI apoptosis necrosis kit. The cells were cultivated for 24 h in cultivation medium in the presence or absence of derivative **11** at the given concentrations. The dot blots are representative of three independent measurements.

Annexin V linked with fluorescein isothiocyanate (FAV), which specifically labels externalized phosphatidylserine on the surface of apoptotic cells, and propidium iodide (PI), which labels DNA in necrotic cells with disrupted plasma membranes [25], were used as markers of apoptosis and necrosis, respectively. Proportions of viable S, R and T cells (i.e., cells that did not bind FAV or PI) after a 24 h incubation period in cultivation medium without derivative **11** (under standard cultivation conditions) consistently exceeded 90% (Figure 2).

The presence of derivative **11** (at a concentration range of 5–25 µM) in the cultivation medium induced an increase in either the proportion of cells labeled with FAV (indicating apoptotic cells) or the proportion of cells labeled by both FAV and PI (indicating cells in late apoptosis). These effects seem to be concentration dependent. The labeling of cells by PI alone (i.e., necrotic cells) was not as pronounced (Figure 2).

### 2.6. Derivative 11 Induced Changes Expression of Bcl-2 and Bax as Well as Expression and Activation of Caspase-3

**Figure 3:**
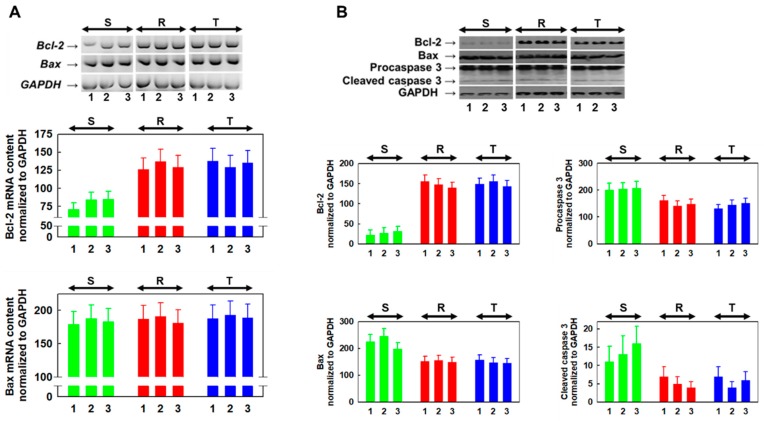
Detection of Bcl-2 and BAX expression as well as caspase-3 expression and activation in S, R and T cells incubated for 24 h in cultivation medium in the absence (1) or presence of derivative **11** at concentrations of 6.5 (2) and 13.0 (3) μM. (**A**) Detection of Bcl-2 and BAX transcripts using RT-PCR in gel (upper part, data are representative of three independent measurements) and its densitometric quantification values (lower part, data are expressed as the mean ± S.E.M. for three measurements). The amount of Bcl-2 transcript detected in S cells differed significantly from the corresponding values for R and T cells (at least at the level of p<0.02) independent of derivative **11**. (**B**) Western blot detection of Bcl-2, Bax, procaspase-3 and caspase-3 in S, R and T cells. The blot records are representative of three independent measurements (upper part), the protein bands were quantified using densitometry (lower part), and the data are expressed as the mean ± S.E.M. for three measurements. The quantities of Bcl-2 protein differ significantly from corresponding values for R and T cells (at least at the level of p < 0.01) independent of derivative **11**. GAPDH mRNA and protein were used as internal standards for RT-PCR and Western blotting.

To study the mechanisms of cell death induced by derivative **11**, we further estimated cell expression of the proapoptotic Bax protein and the antiapoptotic Bcl-2 protein as well as cell content and activation of caspase-3 in S, R and T cells cultivated for 24 h in the absence or presence of 6.5 and 13.0 µM derivative **11** (Figure 3). The expression of Bcl-2 protein is considerably higher in R and T cells than in S cells, as measured by mRNA (Figure 3A) or protein (Figure 3B) levels. In contrast, no similar differences in proapoptotic Bax protein expression between P-gp-negative and P-gp-positive cells were observed either at the mRNA or protein level. The presence of derivative **11** in the cultivation medium did not induce changes in the expression of either protein in S, R or T cells. The massive protein content of procaspase-3 is present in S, R and T cells, and a small proportion of it is proteolytically activated to caspase-3 in all three variants of L1210 cells (Figure 3). However, no statistically significant changes in procaspase-3 content or its cleavage products were detected after S R and T cells were incubated in medium containing derivative **11** at concentrations of 6.5 and 13.0 μM for 24 h.

### 2.7. Effect of Derivative 11 on Cell Cycle Progression of S, R and T Cells

**Figure 4:**
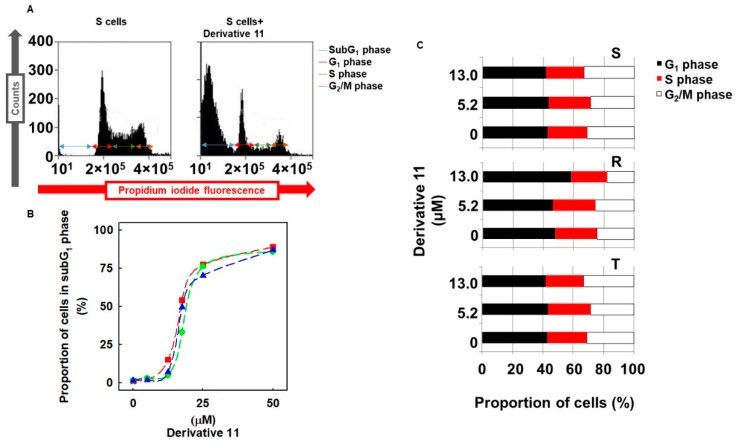
Effect of derivative **11** on cell cycle progression of S, R and T cells. (**A**) Measurement of the cell cycle documented as being in S independent of derivative **11** at a concentration of 13.0 μM. Data are representative of three independent measurements. Similar measurements were made for S, R and T cells in the absence or presence of derivative **11** at concentrations of 0–50 μM, and the resulting data were used for the construction of (**B**,**C**). (**B**) Relative levels of S, R and T cells in subG_1_ phase by the concentration of derivative **11**. Data represent the mean ± S.E.M. of three independent measurements. (**C**) Summary of the effect of derivative **11** on the cell cycle progression of S, R and T cells. Data represent the means from three independent measurements.

The incubation of S, R and T cells for 24 h in the presence of derivative **11** at a concentration of 13.0 μM induced visible cell damage associated with an elevation of the proportion of cells in the subG_1_ phase (Figure 4) when cell cycle progression was monitored by cytometry using DNA cell content detection by PI. Cells in the subG_1_ phase represent the proportion of cells that have enter the progression of programmed cell death and have partially fragmented or degraded DNA. The proportions of S, R and T cells in the subG_1_ phase increased depending on the concentration of derivative **11** (Figure 4B), with an almost identical median effective concentration in the range of 12-18 μM for all three variants of L1210 cells. This finding is consistent with the data documented in Table 2 as obtained using an MTT test of cell viability, in which the median effective concentration was approximately 13.0 μM. According to a previously published recommendation [19], only viable cells, i.e., cells that are not in subG_1_ phase, were used for the analysis of the cell cycle (Figure 4C). Analysis of the cell cycle did not reveal differences between the proportions of S, R and T cells in the respective (G_1_, S or G_2_/M) cell cycle phases. Moreover, the incubation of S, R and T cells in the presence of derivative **11** did not induce an expressed alteration in the proportion of cells in the respective cell cycle phases. An exception of this cell cycle-related behavior, R cells incubated in the presence of derivative **11** at a concentration of 13.0 μM showed an increased proportion of cells in the G_1_ phase (approximately 25%), which was associated with corresponding decreases in the proportion of cells in the S and G_2_/M phases (Figure 4C).

## 3. Discussion

Multiple drug resistance of neoplastic cells leads to reduced cell sensitivity toward different drugs and represents a real obstacle in the effective chemotherapy of neoplastic diseases, including blood malignancies [26]. Until now, diverse but well-understood mechanisms of MDR have been identified [16] of which the efflux activity of overexpressed P-gp in the plasma membrane of neoplastic cells is the most frequently occurring [27]. Therefore, searching for substances that cannot be eliminated from the inner space of cells by overexpressed P-gp is a rational undertaking in medicinal chemistry. For this reason, we have oriented ourselves to study the effective means of cell death induced by phenanthroquinolizidine alkaloid derivatives related to P-gp expression in leukemia cells. We used sets of 13 TQDs, 3 of which are *N*-methylthienoquinolizinium iodides, and 8 BQDs (Table 1) for which the synthesis and chemical characterization have been described elsewhere [13,14,23]. Only derivative **11Ba** was effective from among all the TQD in S cells (with IC_50_ ≈ 130 μM). This derivative did not induce cell death in R and T cells. The corresponding substance A4, with permanent quaternary nitrogen derived from **11Ba** by *N*-methylation, lost cell death effectiveness in S cells, indicating the importance of localized free electron pair on the nitrogen atom of the quinolizidine skeleton. However, permanent positive charge on nitrogen atom may depress ability of substance to enter the cell via passive diffusion, which may be responsible for lack of effectiveness. Derivative **11Bb**, which differs from derivative **11Ba** only in the orientation of the sulfur atom in the thienyl ring, also failed to induce cell death in S cells. Therefore, the proper structural orientation of the thienyl-ring seems to be another crucial feature important for TQD effectiveness in inducing cell death of S cells. It is reasonable to expect that the position of the conjugated π-electron system of the thienyl ring may be important for effecting cell death. Similarities in the conjugated π-electron density at a similar position as in the thienyl-ring of derivative **11Ba** also exist on the benzene ring of BQD derivatives. Derivative **11** exhibits both of the features described above in relation to derivative **11Ba**, i.e., a localized free electron pair on the nitrogen atom of the quinolizidine skeleton and a π-electron system on the benzene ring. This derivative showed the most pronounced effectiveness for inducing cell death in all three variants of L1210 cells, with an IC_50_ ≈ 13 μM independent of P-glycoprotein expression. Derivative **10** differs from derivative **11** by the existence of a formyl group located on carbon atom 3, i.e., in the position directly adjacent to the nitrogen atom of quinolizidine, thus inducing strong delocalization of the free electron pair associated with the nitrogen. This derivative induced only negligible cell death in S cells (IC_50_ ≈ 500 μM) and was ineffective in R and T cells (Table 2). There are also two other effective derivatives, **9** with IC_50_ values 81, 81 and 74 μM for S, R and T cells, respectively, as well as **6b** with IC_50_ values of 192, 385 and 385 μΜ for S, R and T cells, respectively. Both of these derivatives contain the formyl group, described above, in position 3, and substituents (S-methyl xanthate or O-acetyl) in position 10 that could give the derivatives additional ability to cause cell death, most likely by inducing oxidative stress. The property attributed to the localized free electron pair on the nitrogen atom of the quinolizidine skeleton could be parametrized by the respective pK_a_ value of the respective derivative [28]. Therefore, we calculated pKa values for the derivatives **11Ba** and **11** using the online pK_a_ calculator at Instant Cheminformatics Solutions on web (Table 3). Other derivatives with the ability to induce cell death, at least in S cells (**6b**, **9** and **10**), due to the delocalization of free electrons around the nitrogen caused by the existence of a formyl group at position 3 did not have the same acid-base parameterizing characteristics as derivatives **11Ba** and **11**.

**Figure 5:**
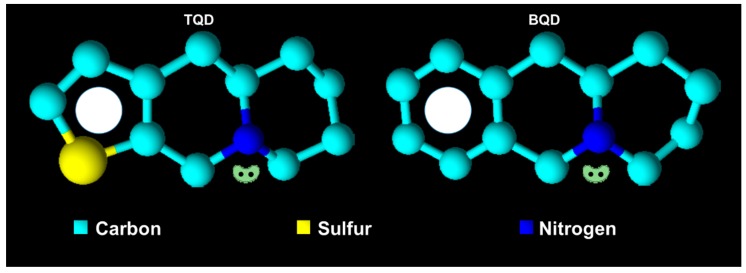
Features important for the effect TQD and BQD on cell death of S, R and T cells: i. Localized free electron pair on the nitrogen atom of derivatives; ii. aromatic character of the terminal left ring with conjugated π electron system; iii. lipophilic character of the substance.

Another important property that could be evaluated when predicting the effectiveness of substances to cause cell death is hydrophobicity, which enables them to enter the inner space of the cell through the plasma membrane by passive diffusion. These properties could be parametrized by the value of the partition coefficient in a two-phase system (frequently water: n-octanol, [29]). For this reason, we calculate the partition coefficient logarithm values in this two-phase system using ACD/ChemSketch for academic and personal use (Advanced Chemistry Development, Inc., Toronto, Ontario, Canada). The most effective at inducing cell death by this parameter, derivative **11** achieved the highest value of logP (3.25) among all derivatives studied (Table 3). Derivative **9** was effective at concentrations below 100 μM and has a logP value of 2.52. The only effective derivative from the TQD group in S cells, **11Ba**, had the second highest logP value (2.93). These facts enable us to propose rules for further design of such derivatives as potential agents for induction of cytotoxic effects in leukemia cells: i. to improve localization of the electron density associated with the nitrogen in the quinolizidine skeleton by introducing electron-donating groups (Figure 5); ii. to potentiate the conjugated π-electron system density on the benzene (thienyl-) part of molecule by introducing electron-donating groups (Figure 5); iii. to modify molecules with structures by improving the hydrophobicity of the substance.

Recently, phenanthroindolizidine alkaloids with similar localized free electron pairs on the nitrogen atom of the indolizidine skeleton and more complex side aromatic systems were shown to induce strong growth inhibitory activity toward a large panel of neoplastic cell lines, including P-gp-overexpressing cells [30].

Derivatives **6b**, **11Ba** and, to a lesser extent, derivative **10** induced cell death more effectively in S than in R and T cells. Differences between the effectiveness of substances on P-gp-negative S and P-gp-positive R and T cells could be caused by the efflux activity of this drug transporter and by simple elimination of substances from the inner space of P-gp-positive cells such that they cannot induce cell death processes [16,27,31]. However, P-gp seems to play a secondary role in silencing apoptosis, and this side effect of P-gp expression is independent of P-gp drug efflux activity [16,31,32]. Thus, this suspected secondary role of P-gp that operates against apoptosis progression may induce differences between the effectiveness of substances that are not substrates of this efflux pump [33].

The most effective cell death-inducing substance we registered was derivative **11**, which is effective at a 10^-5^ M concentration independent of the expression of P-gp. This finding indicated that the presence of P-gp in R and T cells did not protect cells against derivative **11**-induced cell death. Thus, derivative **11** does not seem to be a substrate for P-gp-mediated drug efflux. This derivative induced an elevation of *ABCB1* gene (encoding P-gp) transcription in both P-gp-positive L1210 cell variants (Figure 1). However, this derivative did not alter calcein retention in R and T cells. This finding indicated that derivative **11** does not alter P-gp-mediated substance efflux activity.

In subsequent sets of experiments, we examined the mode of cell death induced by this derivative using a FAV/PI detection kit, which enabled the cytometric quantification of apoptotic (labeled by FAV), necrotic (labeled by PI) and late apoptotic/necrotic cells (labeled by both these markers) [34]. This procedure revealed the elevation of cells labeled with either FAV alone or both FAV and PI and only a negligible proportion of cells labeled with PI alone (Figure 2). This outcome is consistent with apoptotic cell death. Both P-gp positive cell variants (R and T) cultivated in the absence of derivative **11** contained higher levels of antiapoptotic Bcl-2 protein than found in P-gp-negative S cells (Figure 3). The coexpression of Bcl-2 and P-gp confers resistance against the induction of apoptosis in leukemia cells originating from alterations in the lymphoid pathway of hematopoiesis [35]. This difference in Bcl-2 expression also persists in the presence of derivative **11**. We further observed a massive amount of procaspase-3, which was partially cleaved to the active 17 kDa form (Figure 3). This caspase activates caspase-dependent DNase, which fragments DNA as a result of both the intrinsic and extrinsic pathways of caspase-dependent apoptosis [36]. Therefore, our results indicated the readiness of S, R and T cells to initiate apoptosis. However, all three cell variants failed to upregulate this apoptotic marker after treatment with derivative **11**.

During the cultivation of S, R and T cells in medium containing derivative **11**, we observed an increase in the proportion of dead cells in subG_1_ with fragmented/degraded DNA (when cell DNA content was quantified with PI), which is often attributed to initiation and progression of apoptosis [37]. Viable cells after incubation with derivative **11** did not differ significantly in cell proportion by position in the G1, S or G2/M phase of the cell cycle.

## 4. Materials and Methods

### 4.1. Chemicals

In the present paper, sets of previously synthesized and characterized quinolizidine derivatives were tested for cytotoxic effects on P-gp-negative and P-gp-positive L1210 cells (Table 1): i. 13 (epi)- thieno analogs of phenanthroquinolizidines with similar structural motifs as the naturally occurring cryptopleurine and hydroxycryptopleurine [13]; ii. three *N*-methylthienoquinolizinium iodides obtained by *N*-alkylation of corresponding thienoquinolizidines [23]; iii. eight benzo analogs and epi-benzo analogs of bioactive phenanthroquinolizidine alkaloids, which were prepared by asymmetric synthesis as described elsewhere [14].

#### 4.1.1. Components of Cultivation Medium

RPMI 1640 medium with L-glutamine (1 mg/mL), 8% fetal bovine serum, 100,000 units/l of penicillin and 50 mg/l of streptomycin were from Merck in Slovakia (Bratislava, Slovakia) and 1 μg/mL gentamycin was from Thermo Fisher Scientific s.r.o.

#### 4.1.2. Kits

The apoptosis/necrosis kit based on cell labeling with FAV and PI, the ProteomeExtract Subcellular Proteome Extraction Kit were from Calbiochem (San Diego, CA, USA) and GenElute™ Mammalian Total RNA Miniprep Kit (Sigma-Aldrich through Merck in Slovakia, Bratislava, Slovakia)

#### 4.1.3. PCR Primers

The following primers were used for RT-PCR and qRT-PCR: GAPDH F: 5′-TGA ACG GGA AGC TCA CTG G-3′and R: 5′-TCC ACC ACC CTG TTG CTG TA-3′, which produced a 307 bp product; mouse P-gp F: 5′-GGC TGT TAA AGG TAA CTC C-3′ and R: 5′- TGT TCT CTT ATG AAT CAC GTA-3′, which produced a 152 bp product; human P-GP, F: 5′-CTC CTG TCG CAT TAT AGC-3′, R: 5′-AGA CAT GAC CAG GTA TGC-3′, which produced a 151 bp product; Bcl-2 F: 5’-GGC TGG GGA TGA CTT CTC TC-3´, R: 5´-GCA TGCT GGG GCC ATA TAG TT-3´, which produced a 323 bp product; and Bax F: 5´-ATC CAA GAC CAG GGT GGC T-3´, which produced a 197 bp product.

#### 4.1.4. Antibodies

For visualization of protein bands on Western blots, the following antibodies were used: N-20 (sc-493) rabbit polyclonal antibody against Bax, C-2 (sc-7382) mouse monoclonal antibody from Santa Cruz Biotechnology (Dallas, TX, USA); rabbit polyclonal antibody (9662S) against caspase-3 from Cell Signaling Technology Europe (Leiden, The Netherlands); and mouse monoclonal antibody against GAPDH (MAM 374) from Merc in Slovakia. Antimouse recombination antibody (sc-516102) or mouse anti-rabbit affinity purified polyclonal antibody linked with horseradish peroxidase (HRP) were used as secondary antibodies and were purchased from Santa Cruz Biotechnology. HRP signals were visualized using an ECL detection system (GE Healthcare Europe GmbH, Vienna, Austria) and an Amersham Imager 600 (GE Healthcare Europe GmbH, Pittsburgh, PA, USA).

#### 4.1.5. Other Chemicals

MTT ([3-(4,5-dimethyldiazol-2-yl)-2,5 diphenyltetrazolium bromide]), and calcein/AM, verapamil, vincristine, propidium iodide and Triton X-100 were purchased from Merck in Slovakia (Bratislava, Slovakia). RNase A was from Thermo Fisher Scientific s.r.o. (Bratislava, Slovakia). All other chemicals were from Merck in Slovakia (previously Sigma Aldrich).

### 4.2. Cell Culture Conditions

We used three variants of the mouse lymphocytic leukemia cell line L1210: i. P-gp-negative drug-sensitive parental L1210 cells (ACC-123, S) obtained from Leibniz-Institut DSMZ-Deutsche Sammlung von Mikroorganismen und Zellkulturen GmbH (Braunschweig, Germany); ii. P-gp-positive, drug-resistant cells (R) overexpressing P-gp due to selection with vincristine [18]; P-gp-positive, drug-resistant cells (T) overexpressing P-gp due to stable transfection with Addgene plasmid 10957 (pHaMDRwt), and a retrovirus encoding full-length P-gp cDNA [38]). Transfection and cell characterization were completed as described elsewhere [17].

All variants of L1210 cells (S, R and T) were cultured in the RPMI medium described above at 37 °C in a humidified atmosphere with 5% CO_2_. This procedure is termed passage and was repeated three times a week. R cells were periodically passaged each second in the presence of vincristine (250 nM), and were harvested for experiments during the second passage without vincristine. Cell viability was monitored using Countess™ II FL, Invitrogen through Thermo Fisher Scientific s.r.o. R cells were cultured for two passages without VCR prior to the experiments. The expression/activity/localization of P-gp was periodically analyzed by RT-PCR, Western blotting, calcein retention assay and confocal immunocytochemistry [24]). All cell variants (S, R and T) were cultivated in the absence or presence of the respective quinolizidine derivatives at a concentration range of 1-500 μΜ and were used for further examination.

### 4.3. Measurements of Cytotoxic Effects of Quinolizidine Derivatives on S, R and T Cells

S, R and T L1210 cells (5 × 104 cells/well) were incubated with TQD or BQD in a concentration range of 1–500 µM in 96-well culture plates for 48 h. Tested quinolizidine derivatives were added directly to 200 μL of RPMI culture media, and cell viability was assessed using an MTT assay [39]. In brief, after 48 h, the plates were centrifuged for 10 min at 2500 rpm; the cells were resuspended in 200 μL RPMI without fetal bovine serum, and MTT (0.25 mg/mL, i.e., 50 μg per well) was added for 2 h in the dark at 37 °C and then centrifuged for 10 min (3000 rpm); cell sediments were extracted with 150 µL dimethyl sulfoxide (DMSO), and absorbance at 540 nm was measured using the Universal Microplate Spectrophotometer mQuant (BioTek Instruments, Inc., Winooski, VT, USA). Dose-responsiveness curves were fitted according to an exponential decay equation (Eq. (1)) by nonlinear regression as previously described [21]:

N = 100% × exp[ln(0.5) × (c/IC50)](1)

where N represents the percentage (from a control in the absence of drugs) of cell viability after culturing in the presence of the tested derivates at concentration c; IC50 is the concentration of a substance when N = 50%; IC50 values were calculated from three independent measurements. The significance of differences was analyzed using an unpaired Student’s t-test.

### 4.4. Detection of the Effect of Derivative 11 on P-gp, Bcl-2 and Bax Transcription

#### 4.4.1. RT-PCR

Total RNA was isolated from S, R and T L1210 cells using the GenElute™ Mammalian Total RNA Miniprep Kit (Sigma-Aldrich through Merck in Slovakia) according to the manufacturer’s instructions. Reverse transcription was performed using 2 μg of template RNA, Random Hexamer primers (100 pmol) (Thermo Fisher Scientific s.r.o., Waltham, MA, USA) and DEPC-treated water at a total volume of 12.5 µL according to the manufacturer’s protocol.

PCR was performed in a 25 μL total volume containing 4 μL of reaction buffer 250 mM Tris-HCl, 250 mM KCl, 20 mM MgCl2, 50 mM DTT, and 0.5 µL Thermo Scientific™ RiboLock™ RNase Inhibitor, 2 µL dNTP mix, 10 mM each and 1 µL RevertAid H Minus Reverse Transcriptase (all from Thermo Fisher Scientific s.r.o., Waltham, MA, USA). After heating at 95 °C for 5 min to inactivate the reverse transcriptase, the samples were subjected to 35 cycles of denaturation (95 °C for 45 s), annealing (57 °C *Mus musculus* and *Homo sapiens* P-gp and 58 °C GAPDH, Bcl-2 and Bax for 30 s) and extension (72 °C, 90 s), followed by a final extension at 72 °C for 10 min. The PCR products were separated on a 2% agarose gel (Merck in Slovakia) and visualized with GelRed™ nucleic acid gel stain (Biotium, Fremont, CA, USA) using an Amersham™ Imager 600 (GE Healthcare Europe GmbH, Pittsburgh, PA, USA). The data were expressed as the relative level of each mRNA normalized to that of the housekeeping gene *GAPDH*. Statistical significance was analyzed using an unpaired Student’s t-test.

#### 4.4.2. qRT-PCR

Total RNA isolation, reverse transcription and the PCR primers were the same as described for RT-PCR. qPCR was run on a 96-well microtitration plate using a CFX96 TouchTM real-time PCR Detection System (Bio Rad, USA). PCR was run in a 10 μL solution containing 500 ng of cDNA, 5 μL of 2x iTaq Universal SYBR^®^ Green Supermix (Bio-Rad), 1 μL of primer solution at a concentration of 5 μmol/L and 2.5 μL of RNase-free UltraPureTM DEPC-treated water for 39 cycles at 57 °C. The samples were measured in triplicate.

### 4.5. Western Blot Procedures

After incubation, the cells were harvested, and crude membrane fractions were prepared with a ProteomeExtract Subcellular Proteome Extraction Kit according to the manufacturer’s instructions. The proteins from the samples were separated by sodium dodecyl sulfate-polyacrylamide electrophoresis using 10% polyacrylamide gels according to the classic Laemmli [40] protocol and then transferred by electroblotting to a polyvinylidene difluoride (PVDF) membrane (GE Healthcare Europe GmbH). Finally, proteins were detected with specific primary and secondary antibodies linked with horseradish peroxidase. Electroblotting and interaction with antibodies were performed using the classic Towbin protocol [41], and HRP signals were visualized with the ECL system.

### 4.6. Estimation of P-gp Transport Activity by Calcein/AM Retention Assay

P-gp transport activity was measured using a previously described protocol for the calcein retention assay [42]. After cultivation, S, R and T Ll1210 cells were harvested by centrifugation (500× *g*), and 5 × 10^5^ cells were incubated with derivative **11** for 1 h at final concentrations of 5.2 and 13.0 µM. Then, the cells were centrifuged (500× *g*), washed three times with PBS containing 0.2% BSA and then resuspended in 500 μL of the same buffer. Calcein/AM (final concentration 0.1 μmol/L) was added directly to the buffer, and the samples were incubated for 20 min at 37 °C in a CO_2_ incubator. Calcein retention assays were performed in the absence or presence of derivative **11** at final concentrations (5.2 and 13.0 μM). For an inhibitor of P-gp, we used verapamil (final concentration of 10 μM), which was added to the samples together with calcein/AM. Finally, cells were incubated with propidium iodide (final concentration 0.9 μmol/L, Sigma-Aldrich) for an additional 10 min and then washed twice with ice-cold PBS. Fluorescence was measured using an Accuri C6 flow cytometer (BD Bioscience, San Jose, CA, USA). Only viable, propidium iodide-negative cells were counted.

### 4.7. Measurement of Apoptosis/Necrosis Induced by Derivative 11 in S, R and T Cells

Cells (1 × 10^6^/^mL^) were incubated for a 24-h incubation with or without derivative **11** (5–50 µM) under standard culture conditions. The proportions of apoptotic and necrotic cells were then detected using an FAV and PI kit. According to the procedure described by the manufacturer, the cells were washed twice with PBS and gently resuspended in binding buffer (obtained from the manufacturer) containing 0.5 μg/mL FAV. The mixture was then incubated in the dark for 15 min at room temperature and centrifuged (500×*g*, 15 min). The resulting sediments were resuspended in binding buffer, and propidium iodide (final concentration 0.6 μg/mL) was added to each sample, which was analyzed by flow cytometry using an Accuri C6 flow cytometer.

### 4.8. Detection of Cell Proportions in Different Phases of the Cell Cycle

Cells (1 × 10^6^/mL) were incubated for 24 in the absence or presence of derivative **11** (5–50 µM) under standard cultivation conditions. Then, the cells were washed with PBS, resuspended in 0.05% Triton X-100 (Sigma-Aldrich, St. Louis, MO, USA) dissolved in PBS containing 0.1 mg/mL RNase A and incubated for 20 min at 37 °C. The final mixtures were cooled on ice for 10 min, and propidium iodide (40 μg/mL) was added to each sample, which was then incubated on ice for another 30 min. Finally, the specimens were evaluated by flow cytometry on an Accuri C6 flow cytometer.

## 5. Conclusions

We identified derivative **11** as the most effective among those in a set of derivatized quinolizidines. The derivative-induced cell death in L1210 mice lymphocytic cells independent of the expression of P-glycoprotein at a concentration of 13 μM. This derivative is much less effective on the normal fibroblast VERO and BHK-21 cells, for which the effective concentration exceeded 200 μM. Therefore, derivative **11** represents a sufficient starting point for the future design of effective structures.

## Figures and Tables

**Table 1:** Structures of the assayed derivatives.

**(epi)-thieno analogs of phenanthroquinolizidine alkaloids (TQDs)**			
**Derivative**	**Structure**	**Derivative**	**Structure**
**11Ba**^1^ M_w_ = 193.31 g/mol	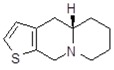	**11Bb**^1^ M_w_ = 192.31 g/mol	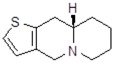
**11Aa**^1^ M_w_ = 207.29 g/mol	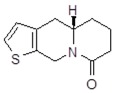	**11Ab**^1^ M_w_ = 207.29 g/mol	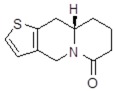
**A4**^2^ M_w_ = 335.25 g/mol	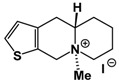	**B10**^2^ M_w_ = 235.25 g/mol	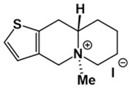
**B9**^2^ M_w_ = 351.25 g/mol	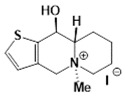	**trans10Bb**^1^ M_w_ = 209.31 g/mol	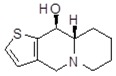
**trans10Aa**^1^ M_w_ = 223.29 g/mol	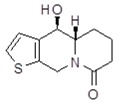	**trans10Ab**^1^ Mw = 223.29 g/mol	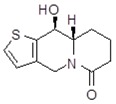
**cis10Aa**^1^ M_w_ = 223.29 g/mol	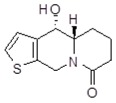	**cis10Ab**^1^ M_w_ = 223.29 g/mol	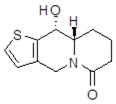
**15a**^1^ M_w_ = 221.28 g/mol	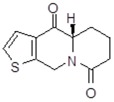	**15b**^1^ M_w_ = 221.29 g/mol	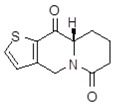
**18Aa**^1^ M_w_ = 263.33 g/mol	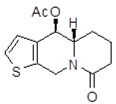	**18Ab**^1^ M_w_=265.33 g/mol	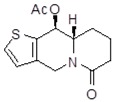
**(epi)benzo analogs of phenanthroquinolizidine alkaloids (BQDs)**			
**5a**^3^ M_w_ = 217,27 g/mol	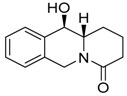	**5b**^3^ M_w_ = 217.27 g/mol	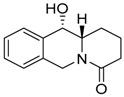
**6b**^3^ M_w_ = 259.31 g/mol	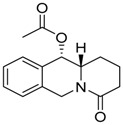	**9**^3^ M_w_ = 307.43 g/mol	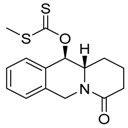
**7a**^3^ M_w_ = 203.29 g/mol	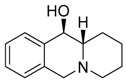	**7b**^3^ M_w_ = 203.29	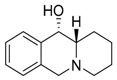
**10**^3^ M_w_ = 201.27 g/mol	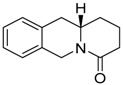	**11**^3^ M_w_ = 187.29 g/mol	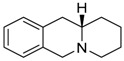

Synthesis and substance characterizations are described elsewhere: 1–[13]; 2–[23], 3–[14].

**Table 2:** The IC_50_ values for the growth inhibition of S, R and T cells by quinolizidine derivatives.

(epi)-thieno analogs of phenanthroquinolizidine			
Derivative	IC_50_ (µmol/l)		
	S	R	T
**11Ba**	130 ± 9	na	na
**11Bb**	na	na	na
**11Aa**	na	na	na
**11Ab**	na	na	na
**A4**	na	na	na
**B10**	na	na	na
**B9**	na	na	na
**trans10Bb**	na	na	na
**trans10Aa**	na	na	na
**trans10Ab**	na	na	na
**cis10Aa**	na	na	na
**cis10Ab**	na	na	na
**15a**	na	na	na
**15b**	na	na	na
**18Aa**	na	na	na
**18Ab**	na	na	na
(epi)-benzo analogs of phenanthroquinolizidine alkaloids			
**5a**	na	na	na
**5b**	na	na	na
**6b**	192 ± 12	385 ± 16	385 ± 19
**7a**	na	na	na
**7b**	na	na	na
**9**	81 ± 5	81 ± 4	74 ± 3
**10**	490 ± 43	na	na
**11**	13,1 ± 2,5	13,4 ± 3,1	12,8 ± 2,7

na–not active.

**Table 3:** Physicochemical properties of effective derivatives.

Derivative		Effective on (μΜ)		pK_a_^1^	Log P^2^
	S	R	T		
**11Ba**	≈130	Na	Na	8.89	2.93 ± 0.35
**6b**	≈190	≈385	≈385	-	1.05 ± 0.62
**9**	≈80	≈80	≈74	-	2.52 ± 0.69
**10**	≈490	Na	Na	-	1.62 ± 0.56
**11**	≈13	≈13	≈13	9.20	3.25 ± 0.32

^1^ Calculated using the online pK_a_ calculator Instant Cheminformatics Solutions on web. ^2^ Calculated using ACD/ChemSketch for academic and personal use (Advanced Chemistry Development, Inc. Toronto, Ontario, Canada).
